# Antioxidant, Antibacterial and Antibiofilm Activity of Nanoemulsion-Based Natural Compound Delivery Systems Compared with Non-Nanoemulsified Versions

**DOI:** 10.3390/foods12091901

**Published:** 2023-05-06

**Authors:** Bruno Dutra da Silva, Denes Kaic Alves do Rosário, Luiz Torres Neto, Carini Aparecida Lelis, Carlos Adam Conte-Junior

**Affiliations:** 1 Analytical and Molecular Laboratorial Center (CLAn), Institute of Chemistry (IQ), Federal University of Rio de Janeiro (UFRJ), Cidade Universitária, Rio de Janeiro 21941-909, Brazil; brunodutrads@gmail.com (B.D.d.S.); deneskaic@gmail.com (D.K.A.d.R.); luiz-torres-neto@hotmail.com (L.T.N.); carinilelis@gmail.com (C.A.L.); 2 Center for Food Analysis (NAL), Technological Development Support Laboratory (LADETEC), Federal University of Rio de Janeiro (UFRJ), Cidade Universitária, Rio de Janeiro 21941-598, Brazil; 3 Laboratory of Advanced Analysis in Biochemistry and Molecular Biology (LAABBM), Department of Biochemistry, Federal University of Rio de Janeiro (UFRJ), Cidade Universitária, Rio de Janeiro 21941-909, Brazil; 4 Graduate Program in Food Science (PPGCAL), Institute of Chemistry (IQ), Federal University of Rio de Janeiro (UFRJ), Cidade Universitária, Rio de Janeiro 21941-909, Brazil; 5 Nanotechnology Network, Carlos Chagas Filho Research Support Foundation of the State of Rio de Janeiro (FAPERJ), Rio de Janeiro 20020-000, Brazil; 6 Department of Food Engineering, Center for Agrarian Sciences and Engineering, Federal University of Espírito Santo (UFES), Alto Universitário, S/N Guararema, Alegre 29500-000, Brazil

**Keywords:** *Origanum vulgare*, essential oil, carvacrol, thymol, foodborne illness, nanotechnology

## Abstract

This study aimed to develop nanoemulsions with a focus on improving the bioactivity of oregano essential oil (OEO), carvacrol and thymol for possible food applications. Nanoemulsions were prepared with acoustic cavitation using ultrasound. The nanodroplets had average diameters of 54.47, 81.66 and 84.07 nm for OEO, thymol and carvacrol, respectively. The main compound in OEO was carvacrol (74%), and the concentration in the nanoemulsions was 9.46 mg/mL for OEO and the isolated compounds. The effects of droplet size reduction on antioxidant, antibacterial and antibiofilm activity were evaluated. Regarding antioxidant activity, the nanoemulsions performed better at the same concentration, with inhibitions >45% of the DPPH radical and significant differences compared with their non-nanoemulsified versions (*p* < 0.05). The nanoemulsions’ minimum inhibitory concentration (MIC) and non-nanoemulsified compounds were evaluated against foodborne pathogens with inhibition ranges between 0.147 and 2.36 mg/mL. All evaluated pathogens were more sensitive to nanoemulsions, with reductions of up to four times in MIC compared with non-nanoemulsified versions. *E. coli* and *S*. Enteritidis were the most sensitive bacteria to the carvacrol nanoemulsion with MICs of 0.147 mg/mL. Concerning antibiofilm activity, nanoemulsions at concentrations up to four times lower than non-nanoemulsified versions showed inhibition of bacterial adhesion >67.2% and removal of adhered cells >57.7%. Overall, the observed effects indicate that droplet size reduction improved the bioactivity of OEO, carvacrol and thymol, suggesting that nanoemulsion-based delivery systems for natural compounds may be alternatives for food applications compared with free natural compounds.

## 1. Introduction

Essential oil nanoemulsions are colloidal dispersions at the nanometer scale where the oil is dispersed in a continuous aqueous phase, being adsorbed by a surfactant that reduces the interfacial tension. Generally, droplet diameter in nanoemulsions should be <200 nm to exhibit improved physicochemical properties and kinetic stability [1]. The increase in surface area due to the nanometric size of the droplets provides a greater possibility of chemical interaction of bioactive compounds with targets in the bacterial cell or free radicals. Different methods of preparing nanoemulsions to evaluate physicochemical and bioactive properties have been studied in recent years [2,3]. These studies are necessary to reinforce the knowledge about improving physicochemical properties in nanoemulsions of essential oils. However, the differences between the formulations and direct applications in the food matrix make it challenging to understand the mechanism of action and standardize the preparation technique in order to increase the bioactivity of the natural compounds.

A meta-analysis by da Silva et al. (2022) [4] proposed that nanoemulsions bioactivity is related to droplet size reduction and mainly to the surfactant/oil ratio (SOR), which should be ideal for maintaining the bioactive hydrophobic core. The development of nanoemulsions using ultrasound has gained interest due to the low cost of implementation. The acoustic cavitations of the device create pressure variations that cause the microbubbles in the system to collapse. The turbulence created by the collapse is responsible for the droplet’s breakup and size reduction [5].

*Origanum* species are perennial herbs cultivated worldwide, composed of 43 species and 18 hybrids and widely used in food preparation. The chemical composition of oregano essential oil (OEO) can vary depending on growing conditions, climate and geographic regions. Still, the major compounds found generally include thymol and carvacrol in different concentrations [6,7]. OEO and its major compounds are recognized as safe for consumption and regulated by the Federal Drug Administration (FDA). Currently, consumers are looking for a more natural diet, increasing the consumption of foods without traditional preservatives, known as “Clean Label” products [8,9]. Several studies highlighted OEO’s antimicrobial and antioxidant activity in vitro and in food matrices [10,11]. However, using essential oils in food has limitations, including the chemical interaction of essential oil constituents with the food matrix and water insolubility [12]. Nanoemulsions have recently been suggested in food science as an alternative to overcome the limitations of essential oils in food matrices. The number of papers on natural compounds nanoemulsified has increased in recent years, but there is still a gap regarding the preparation of nanoemulsions with improved bioactive properties [13]. The lack of knowledge about size reduction with improvement in droplet properties has hampered conclusive answers about using nanoemulsions as an alternative to free natural compounds.

Different studies showed results with greater, equal or less antioxidant and antibacterial activities of nanoemulsions compared with their non-nanoemulsified versions [14]. There is a gap in the standardization of nanoemulsion development parameters associated with the bioactive effects of natural compounds. The chemical composition of the nanodroplet is also fundamental in these characteristics and can be altered to enhance the application’s action [15]. Using nanoemulsion can reduce the limiting impacts of pure oil and improve antioxidant and antibacterial properties. Focusing on understanding the knowledge gaps about the bioactivity of nanoemulsion-based delivery systems, the present study aimed to develop a nanoemulsion with OEO, carvacrol and thymol using ultrasound and compare the antioxidant, antibacterial and antibiofilm activity with its non-nanoemulsified versions.

## 2. Materials and Methods

### 2.1. Materials

The OEO originated in Moldova and was purchased from Ferquima Ltd. (São Paulo, Brazil). The essential oil was extracted using steam distillation, and the main components were carvacrol (74%), *p*-cymene (5%) and thymol (2%), according to the manufacturer’s technical report. The carvacrol (CAR), thymol (THY) and 2,2-diphenyl-1-picrylhydrazyl (DPPH) were purchased from Sigma-Aldrich (São Paulo, Brazil). Tween 80 (ISOFAR, Duque de Caxias, Brazil) was used as a surfactant. *Escherichia coli* ATCC 25922, *Salmonella enterica* subsp. *enterica* serovar Enteritidis ATCC 13076 and *Staphylococcus aureus* ATCC 13565 were obtained from the culture bank of Fundação Oswaldo Cruz (FIOCRUZ, Rio de Janeiro, Brazil). Strains of *Listeria monocytogenes* ATCC 7644 were obtained from the Federal University of Espírito Santo (UFES, Brazil).

### 2.2. Nanoemulsion Development

A coarse emulsion (50 mL) was prepared by adding 1% OEO, CAR or THY with 2.9% Tween 80, based on an improved formulation proposed by da Silva et al. (2023) [16]. The remaining volume consisted of ultrapure water (Mili-Q IQ 7005, Merck, Darmstadt, Germany). The emulsion was prepared in Ultraturrax ( T10, IKA^®^, Shanghai, China) for 10.000 rpm at 5 min. The Ultraturrax had an output power of 75 W and a dispersion shaft (S10N-5G, IKA, Shangai, China) with the following specifications: rotor diameter = 5 mm; stator diameter = 3.8 mm; length of spreading shaft = 178 mm. The dispersant shaft’s immersion depth in the emulsion was 35 mm.

Subsequently, the emulsion was sonicated at 157.5 W for 4.9 min in ultrasound (VC-750 Ultrasonic Processor, 20 kHz, 750 W) with a 19 mm diameter titanium probe (Sonics, Materials Inc., Newtown, CA, USA), which was immersed 60 mm into the emulsion. The effective power of ultrasound was measured using calorimetry (Power = 13.83 W) (Equation (1)), and the energy dissipated in the fluid was determined by the acoustic density energy (AED = 81.3 kJ/mL) (Equation (2)) [17]. The emulsion was cooled by an ice bath during processing to avoid excessive temperature. The temperature did not exceed 20 °C. After preparation, the nanoemulsions were stored at 4 °C.

(1) $$Power\;(W)=mC_{p}\left.\left(\frac{\partial T}{\partial t}\right.\right)$$

(2) $$AED\;(kJ/mL)=\frac{Power\left(W\right)\cdot process\;time\;(s)}{Sample\;(mL)}$$

wherein *m* = mass of solvent (g), C_p_ = solvent specific heat (kJ/g), *∂T* = increased temperature (°C), *∂t* = sonication time (s).

### 2.3. Physicochemical Properties of Nanoemulsions

The droplet size (DS), polydispersity index (PDI) and zeta potential (ZP) of oregano essential oil (*n*OEO), carvacrol (*n*CAR) and thymol nanoemulsions (*n*THY) were determined in Dynamic Light Scattering (Zetasizer Nano^®^, Model 590, Malvern Instruments, Malvern, UK). Samples were diluted 1:10 in ultrapure water, and values were determined by averaging three measurements at 25 °C. Data were reported as mean droplet diameter ± standard deviation (*n* = 3). Each sample was analyzed for size and PDI in a disposable polystyrene cell (DTS0012, Malvern Instruments, Malvern, UK). Disposable capillary cells (DTS 1060, Malvern Instruments, Malvern, UK) were used to measure the zeta potential. The pH of the nanoemulsions was measured with a pH meter (FiveEasy, Mettler Toledo, Jurong, Singapore).

### 2.4. Antioxidant Activity

The reduction capacity of the 2,2-diphenyl-1-picrylhydrazyl (DPPH) radical was evaluated based on the method described by Rufino et al. (2007) [18] and Baj et al. (2018) [19]. OEO, CAR and THY concentrations were standardized to avoid bias in the assay to equal the concentration in the nanoemulsions (9.46 mg/mL). In the dark, 100 μL of the OEO, CAR, THY and their respective nanoemulsified versions were added to 3.9 mL of DPPH solution (0.06 mM) diluted in methanol. The mixture was homogenized and left to rest for 90 min. After the reaction period, the absorbances of the samples were read in a spectrophotometer (UV-1900i, Shimadzu, Kyoto, Japan) at 515 nm, and a methanol solution with the DPPH reagent was used as a control. The DPPH radical inhibition rate was determined using Equation (3):

(3) $$Inhibition\left(\%\right)=\left(\frac{{Abs}_{control}-{Abs}_{sample}}{{Abs}_{control}}\right)\times 100$$

### 2.5. Antibacterial Activity

#### 2.5.1. Preparation of Bacterial Suspensions

*Escherichia coli*, *Salmonella* Enteritidis, *Staphylococcus aureus* and *Listeria monocytogenes* were selected for testing because they are the most important pathogens associated with foodborne illness. The pathogens investigated in the present study were activated twice in Brain Heart Infusion broth (Kasvi, Madrid, Spain), incubated for 24 h at 37 °C. After this, the pathogens were isolated on different selective agars: EMB agar (Kasvi, Madrid, Spain) for *E. coli* ATCC 25922; XLD agar (Kasvi, Madrid, Spain) for *S*. Enteritidis ATCC 13076; Listeria Oxford agar (Neogen, Manchester, England) for *L. monocytogenes* ATCC 7644; and Baird Parker agar (Kasvi, Madrid, Spain) supplemented with egg yolk tellurite for *S. aureus* ATCC 13565. Colonies of isolated bacteria were transferred to test tubes containing 5.0 mL of sterile saline solution at 0.85% (*m*/*v*). Turbidity was compared with a standard barium sulfate solution (0.5 MacFarland standard), which corresponds to an approximate 8 log CFU/mL concentration according to the Clinical and Laboratory Standards Institute guidelines [20]. The bacterial counts were confirmed at the time of testing on Plate Count agar (PCA, Kasvi, Madrid, Spain).

#### 2.5.2. Minimum Inhibitory Concentration (MIC)

The MIC for each pathogen was determined in a microdilution assay in sterile 96 U-bottom well microtiter plates (OLEN, Shanghai, China) according to the method described by Wiegand et al. (2008) [21]. Final concentrations of 2.36, 1.18, 0.59, 0.29, 0.147, 0.07 and 0.035 mg/mL of OEO, CAR, THY and their respective nanoemulsified versions were obtained from a stock solution diluted with sterile Mueller Hinton broth at a concentration of 9.46 mg/mL. OEO, CAR and THY were diluted in Mueller Hinton broth +0.5% Tween 80 due to insolubility in water at the concentrations evaluated. An amount of 100 μL of each bacterial suspension was added to each well in triplicate to reach 5.5 log CFU/mL. Bacterial counts were confirmed on PCA agar at the time of testing. The microtiter plates were incubated at 37 °C for 24 h, and the MIC was considered the lowest concentration that prevented visible bacterial growth in the microtiter plate wells. Wells containing only Mueller Hinton broth +0.5% Tween 80 and Mueller Hinton broth +0.5% Tween 80 with bacterial inoculum were used for sterility and growth control, respectively. The antibacterial activity of Tween 80 was also evaluated at the same concentrations as the natural compounds.

#### 2.5.3. Bacterial Adhesion

The antiadhesion activity of OEO, CAR, THY and their respective nanoemulsified versions were evaluated against strains of *E. coli* ATCC 25922, *S*. Enteritidis ATCC 13076, *S. aureus* ATCC 13565 and *L. monocytogenes* ATCC 7644 based on the crystal violet staining assay in sterile 96 U-bottom well microtiter plates used by Pejčić et al. (2020) [22]. Serial decimal dilutions were prepared from a standardized bacterial suspension of 8 log CFU/mL to achieve a final bacterial concentration of 6 log CFU/mL in the microplate wells. A total of 100 µL aliquots of the bacterial suspensions were individually inoculated into microplate wells with 100 µL OEO, CAR, THY and their respective nanoemulsified versions diluted in Mueller Hinton broth to achieve subinhibitory concentrations (1/2 MIC). OEO, CAR and THY were diluted in Mueller Hinton broth +0.5% Tween 80 due to insolubility in water at the concentrations evaluated. The plates were at 37 °C for 48 h for microbial adhesion. Wells containing Mueller Hinton broth +0.5% Tween 80 (*v*/*v*) and inoculum were used as growth controls. Wells containing Mueller Hinton broth +0.5% Tween 80 (*v*/*v*) were used as sterility controls.

After incubation, the contents of the microtiter plates were removed, and the wells were washed twice with sterile saline 0.85% (*w*/*v*) to remove planktonic cells. Then the plates were dried in an oven at 60 °C for 45 min. The wells were stained with 200 µL of 0.5% crystal violet solution (*w*/*v*) and allowed to rest for 20 min. After this, the wells were washed with sterile distilled water and decolorized with 200 µL of 96% ethyl alcohol (*v*/*v*) for 45 min. The contents of the wells were transferred to a new microtiter plate, and the absorbance (ABS) of the solutions was measured at 570 nm in a microtiter plate reader (Victor™ X4, Perkin Elmer, Waltham, MA, USA). Wells containing only ethyl alcohol were used as a blank. The values obtained were converted into a percentage of inhibition of adhesion (Equation (4)) and compared with bacterial adhesion of the growth control.

(4) $$Inhibition\;of\;adhesion\left(\%\right)=\frac{{ABS}_{control}-{ABS}_{sample}}{{ABS}_{control}}\times 100$$

#### 2.5.4. Removal of Adhered Cells

The removal of adhered cells exposed to natural compounds was based on a study by Pejčić et al. (2020) [22]. Aliquots of 100 µL bacterial suspensions standardized to 6 log CFU/mL were individually inoculated into the wells of the 96-well microtiter plates containing 100 µL Mueller Hinton broth. Microtiter plates were incubated at 37 °C for 48 h. Fresh Mueller Hinton broth was added to all wells of the microtiter plates every 24 h. After adhesion time, the contents of the wells were removed and 200 µL of OEO, CAR, THY and their respective nanoemulsified versions at 2× MIC dissolved in Mueller Hinton broth were added individually to the microplate wells. The microplates were again incubated for 24 h at 37 °C. The crystal violet staining assay was performed after incubation, as described in item 2.6.1. The percentage of adhered cell removal was determined according to Equation (5).

(5) $$Removal\;of\;adhered\;cells\left(\%\right)=\frac{{ABS}_{control}-{ABS}_{sample}}{{ABS}_{control}}\times 100$$

### 2.6. Statistical Analysis

Data were recorded as mean ± standard deviation (SD) of three independent experimental replicates (*n* = 3). One-way ANOVA followed by Duncan’s test was performed to determine differences between treatments (OEO, *n*OEO, CAR, *n*CAR, THY and *n*THY), considering a significance level of 5%. These analyses were performed in XLSTAT software (Version 2021.5, Addinsoft, New York, NY, USA).

## 3. Results and Discussion

### 3.1. Droplet Properties

The average droplet size (DS), polydispersity index (PDI), zeta potential (ZP) and pH of the nanoemulsions are shown in Table 1. All nanoemulsions showed ideal diameters (<100 nm), and *n*OEO had the smallest particle size (*p* < 0.05). Carvacrol and thymol are isomeric terpenoids, which explains the similar sizes obtained for the two compounds. Carvacrol (~74%) and thymol (~2%) were present in the essential oil composition of *Origanum vulgare.* However, the other compounds present in the essential oil (~24%) may have influenced its physicochemical properties, such as polarity and viscosity. These changes in chemical composition can affect the shear and surfactant/oil interaction, which impacts the final droplet size. The PDI of the nanoemulsions was below <0.25, a value considered ideal for reducing instability phenomena caused by droplet kinetics over time. The *n*CAR presented the most homogeneous system among the evaluated samples (*p* < 0.05). For the ZP, ideal values for the prevalence of electrostatic repulsion are considered when the electrical charge of the drop is above +30 mV or below −30 mV [23]. The values of the present study are close to −10 mV and are mainly influenced by the surfactant.

Converting a micrometer-scale emulsion into a nanoemulsion requires a significant increase in the liquid–liquid interface. The increase in surface area means an increase in Gibbs free energy in the system, so the process cannot proceed spontaneously without adding external energy [24]. In the present study, the surfactant/oil ratio was approximately 3:1, which allowed droplet size reduction (<100 nm) with milder processing conditions, such as time and ultrasound power. The addition of surfactant reduces tension at the oil/water interface and decreases the pressure difference between the inside and outside of the drop, called Laplace pressure. This pressure acts as a resistance to any stress applied to break up the droplet. Some studies have focused on increasing process time and applied power to overcome this interfacial energy. Ozogul et al. (2020) [25] obtained droplet sizes of 204 nm for a thyme essential oil nanoemulsion (72% carvacrol) with 500 W ultrasound power, 15 min of sonication and SOR of 1:10. Alternatively, Sepahvand et al. (2021) [26] obtained sizes of 86.39 nm in a thymol nanoemulsion with low powers (100 W) and process time (4 min) using SOR 2:1, similar to the present study.

### 3.2. Antioxidant Activity

**Figure 1:**
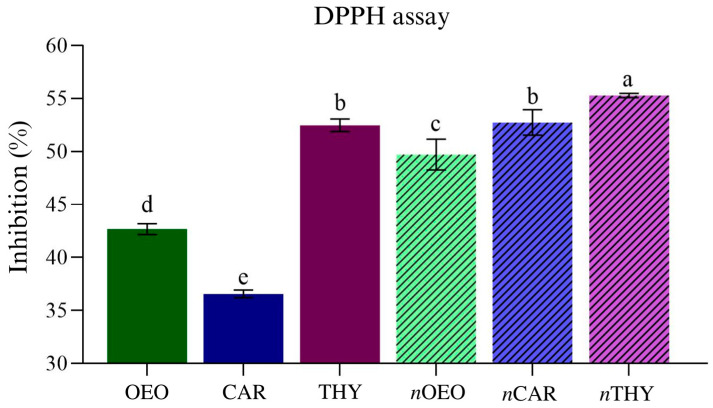
Reducing capacity by 2,2-diphenyl-1-picrylhydrazyl radical inhibition (DPPH) radical. OEO = Origanum *vulgare* essential oil; CAR = carvacrol; THY = thymol; their nanoemulsions, respectively, are *n*OEO, *n*CAR and *n*THY. Different lowercase letters between treatments indicate significant differences according to Duncan’s test (*p* < 0.05).

The reducing capacity of the nanoemulsions and their non-nanoemulsified versions are shown in Figure 1. Tween 80 did not show considerable antioxidant activity at the same concentrations as the natural compounds evaluated (9.46 mg/mL). All nanoemulsions were significantly better than the non-nanoemulsified versions (*p* < 0.05). *n*THY showed the best reducing capacity, reducing 55.3% of the DPPH radical. However, non-nanoemulsified carvacrol showed the lowest antioxidant activity. The improved antioxidant activity of the nanoemulsions observed in the present study may be related to the increased surface area (surface/volume ratio) of the suspended droplets. This effect facilitates the contact of bioactive compounds with free radicals, enabling a more effective electron transfer mechanism than non-nanoemulsified versions [27].

Thymol and carvacrol are isomeric oxygenated monoterpenes with a phenolic and hydroxyl structure that scavenges reactive oxygen species and free radicals by transferring electrons or hydrogen atoms [9]. However, the mechanism of antioxidant action between the compounds may vary due to the position of the hydroxyl in the molecule, as reported in some studies [28]. In addition, it was possible to observe in the present study that the mixture of compounds present in *Origanum vulgare* essential oil with a predominance of carvacrol (~74%) improved its antioxidant effect compared with isolated carvacrol.

The results of the present study agree with previous studies with nanoemulsions of essential oils or isolated compounds. Lou et al. (2017) [29] also reported the DPPH radical reducing capacity for Citrus medica essential oil (50% D-limonene) and its nanoemulsion, with 44.3% and 72.4% free radical inhibition, respectively. Similar results for Mentha spicata *L. essential* oil nanoemulsion (79% carvone) compared with its non-nanoemulsified version were also observed by Zamaniahari et al. (2022) [30].

### 3.3. Minimum Inhibitory Concentration (MIC)

The inhibitory potentials of *n*OEO, *n*CAR, *n*THY and their non-nanoemulsified versions against foodborne pathogens are shown in Table 2. Tween 80 did not show considerable antibacterial activity at the same concentrations as the natural compounds evaluated (MIC > 9.46 mg/mL). For all bacteria, the nanoemulsions showed lower concentrations of inhibition than their non-nanoemulsified versions, indicating that the droplet size reduction improved the antibacterial activity of the system. Overall, *n*CAR presented the best results, with lower values of inhibitory concentrations, considering the four bacteria evaluated, followed by *n*THY and *n*OEO.

*S.* Enteritidis and *E. coli* were the most sensitive bacteria to all evaluated nanoemulsified versions. *S. aureus* and *L. monocytogenes* had the highest inhibitory concentration reductions (4×) for *n*OEO and *n*THY. Gram-negative bacteria are generally reported to be less sensitive to essential oils because they have an outer membrane composed mainly of lipopolysaccharides that make it difficult for oil to penetrate the cell. However, in the present study, gram-negative bacteria were more sensitive than gram-positive bacteria. Studies in the literature also found the same behavior for several essential oils [31]. The mechanism for combating induced stress in the cell membrane may vary between bacterial species, which could account for the variation observed in the present study [32].

Causing damage to a cell structure is the main mechanism of action of essential oils, as they can cause leakage of intracellular content due to increased membrane permeability. The hydrophobic nature of essential oils is responsible for their ability to interact with the cytoplasmic membrane. In addition, increasing membrane permeability facilitates the incorporation of oil components into the cell that can interact with hydrophobic sites of intracellular compounds, promoting vital changes in the bacterial system [33].

The improved solubility of nanoemulsions favors an increase in the droplet gradient of essential oil and bioactive compounds in the aqueous phase, where bacterial cells are present. Nanodroplets can also permeate by passive diffusion through the bacterial cell up channels called porins. All these mechanisms occur simultaneously, which favors the antibacterial activity of nanoemulsions. In addition, the reduction in the size of the droplets leads to an increase in the surface area, which increases the probability of contact of the bioactive compounds with the cytoplasmic membrane and the sites of intracellular interaction [16]. Similar results were observed by Jiménez et al. (2018) [34] with an ultrasound-prepared nanoemulsion (*Cinnamomum zeylanicum* and *Piper nigrum*) that showed superior activity to the pure oil against gram-positive and gram-negative bacteria. Even with larger diameters (171.88 nm) than those obtained in the present study, Moghimi et al. (2018) [35] obtained improved antibacterial activity for the *Thymus daenensis* essential oil nanoemulsion (70% thymol) prepared with ultrasound against *Acinetobacter baumannii*, reinforcing that the droplet diameter reduction improves the antibacterial activity of the oil.

### 3.4. Antiadhesion Activity

**Figure 2:**
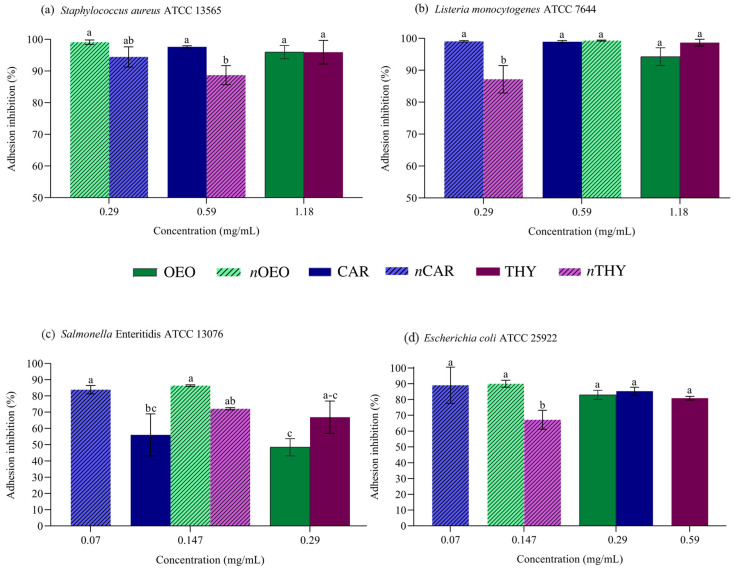
Antiadhesion activity of *Origanum vulgare* essential oil (OEO), carvacrol (CAR), thymol (THY) and their nanoemulsions, respectively (*n*OEO, *n*CAR, *n*THY) against *Staphylococcus aureus* (**a**), *Listeria monocytogenes* (**b**), *Salmonella* Enteritidis (**c**) and *Escherichia coli* (**d**). Different lowercase letters between treatments indicate significant differences according to Duncan’s test (*p* < 0.05).

Subinhibitory concentrations were chosen so there was no influence on bacterial growth, and the antiadhesion effects are shown in Figure 2. The inhibition of bacterial adhesion ranged from 48.5 to 99.3%. Most nanoemulsions had better or similar results than their non-nanoemulsified versions, even at lower concentrations. The exception was thymol, which was superior to its nanoemulsion for *S. aureus*, *E. coli* and *L. monocytogenes* (*p* < 0.05). However, the *n*THY four times less concentrated than the isolated compound showed an average inhibition above 67.1% of bacterial adhesion.

Adhesion is a critical step in which bacteria attach to surfaces and produce a polymeric matrix composed mainly of polysaccharides and glycoproteins to arrange themselves favorably to the surrounding environment. The biofilms that can be formed after adhesion benefit the bacterial community, such as by providing protection from antibacterial compounds and controlled nutrient flow [36].

The effects observed at subinhibitory concentrations on bacterial adhesion may be related to the stress suffered by the bacteria when in contact with essential oils and isolated compounds. In subinhibitory conditions, the mechanism of antibacterial action occurs at a lower intensity, and the increase in membrane permeability without the ability to cause cell lethality causes cell stress [37]. Any change in optimal environmental conditions influences the stress on a microorganism. In an adverse environment, bacteria begin to express genes that fight stress, impacting the expression of bacterial adhesion genes, such as pili, fimbriae and exopolysaccharides. The extent of this stress determines whether the bacteria are killed, the growth is inhibited or the latency time and growth rate are reduced [32].

Another hypothesis evaluated in recent years is that the production of flagella, fimbriae, pili and exopolysaccharides regulated by quorum sensing can be affected by essential oils. Studies on quorum sensing indicate significant inhibition of adhesion-associated gene expression when bacterial cells are exposed to essential oils and isolated compounds at subinhibitory concentrations [38]. In the present study, both mechanisms may act simultaneously according to the concentration of the compound in contact with bacterial cells. Similar results were observed with subinhibitory concentrations for a nanoemulsified essential oil from *Thymus daenensis* against multidrug-resistant *Acinetobacter baumannii* and a *Satureja khuzistanica* essential oil nanoemulsion against *Pseudomonas aeruginosa* adhesion [35,39].

### 3.5. Removal of Adhered Cells

**Figure 3:**
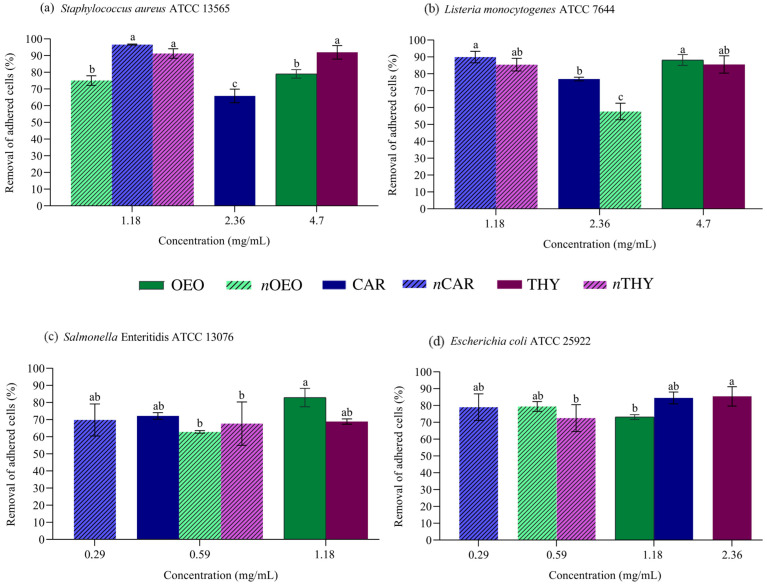
Cell removal activity by *Origanum vulgare* essential oil (OEO), carvacrol (CAR), thymol (THY) and their nanoemulsions, respectively (*n*OEO, *n*CAR, *n*THY) against *Staphylococcus aureus* (**a**), *Listeria monocytogenes* (**b**), *Salmonella* Enteritidis (**c**) and *Escherichia coli* (**d**). Different lowercase letters between treatments indicate significant differences according to Duncan’s test (*p* < 0.05).

The adhesion and production of biofilms contribute to microbial colonization on food contact surfaces under different environmental conditions, which is undesirable in the food industry because it jeopardizes food safety [40]. The removal of adhered bacterial cells exposed to different concentrations of natural antimicrobials is presented in Figure 3. The removal of adherent cells ranged from 57.7 to 96.7%. Differences between the nanoemulsions and their non-nanoemulsified versions were observed (*p* < 0.05). For some bacteria, the nanoemulsions were less effective in removing attached cells than their respective non-nanoemulsified versions, such as *n*THY for *E. coli* and *n*OEO for *S.* Enteritidis and *L. monocytogenes*. However, it is worth mentioning that even at concentrations two to four times lower than their non-nanoemulsified versions, the nanoemulsions showed removal of adhered cells above 57.7%.

Nanoemulsions are expected to effectively penetrate the polymeric matrix of biofilms to contact the cytoplasmic membrane of bacteria due to their small particle size and strong curvature, which justifies the application of nanotechnology to enhance the bioactivity of essential oil [39].

## 4. Conclusions

Nanometric droplets smaller than 100 nm with low polydispersity could be obtained using ultrasound at low power and sonication times. The surfactant/oil ratio (SOR) was crucial to achieving the desired droplet diameters. The preparation of nanoemulsion-based delivery systems improved the antioxidant and antibacterial activity of *Origanum vulgare* essential oil, carvacrol and thymol. Furthermore, nanoemulsions at concentrations up to four times lower than non-nanoemulsified versions showed greater effects in inhibiting bacterial adhesion. The effects observed in this study may solve limitations in developing nanoemulsions using ultrasound with enhanced bioactive properties. In addition, the nanoemulsions developed in the present study can be considered natural alternatives to guarantee the quality and microbiological safety of foods.

## Figures and Tables

**Table 1:** Physicochemical characteristics of the nanoemulsions.

	*n*OEO	*n*CAR	*n*THY
DS (nm)	54.47 ± 0.43 ^a^	81.66 ± 3.95 ^b^	84.07 ± 0.77 ^b^
PDI	0.24 ± 0.01 ^b^	0.17 ± 0.02 ^a^	0.24 ± 0.01 ^b^
ZP (mV)	−8.42 ± 0.74 ^a^	−11.99 ± 0.45 ^b^	−12.77 ± 0.84 ^b^
pH	6.03 ± 0.02 ^a^	6.12 ± 0.03 ^a^	6.03 ± 0.01 ^a^

Values represent mean ± standard deviation. *n*OEO = *Origanum vulgare* essential oil nanoemulsion; *n*CAR = carvacrol nanoemulsion; *n*THY = thymol nanoemulsion; DS = droplet size; PDI = polydispersity index; ZP = zeta potential. Different lowercase letters between treatments indicate significant differences according to Duncan’s test (*p* < 0.05).

**Table 2:** Minimal inhibitory concentrations of nanoemulsions and non-nanoemulsified versions against foodborne pathogens (mg/mL).

Bacteria	OEO	*n*OEO	CAR	*n*CAR	THY	*n*THY
*Escherichia coli*	0.59	0.29	0.59	0.147	1.18	0.29
*Salmonella* Enteritidis	0.59	0.29	0.29	0.147	0.59	0.29
*Staphylococcus aureus*	2.36	0.59	1.18	0.59	2.36	0.59
*Listeria monocytogenes*	2.36	1.18	1.18	0.59	2.36	0.59

OEO = *Origanum vulgare* essential oil; *n*OEO = *Origanum vulgare* essential oil nanoemulsion; CAR = carvacrol; *n*CAR = carvacrol nanoemulsion; THY = thymol; *n*THY = thymol nanoemulsion.

## Data Availability

The data presented in this study are available on request from the corresponding author.
